# Profile of adult and pediatric neurocysticercosis cases observed in five Southern European centers

**DOI:** 10.1007/s10072-016-2606-x

**Published:** 2016-05-18

**Authors:** Lorenzo Zammarchi, Andrea Angheben, Federico Gobbi, Giorgio Zavarise, Ana Requena-Mendez, Valentina Marchese, Carlotta Montagnani, Luisa Galli, Zeno Bisoffi, Alessandro Bartoloni, Jose Muñoz

**Affiliations:** Clinica Malattie Infettive, Dipartimento di Medicina Sperimentale e Clinica, Università Degli Studi di Firenze, Largo Brambilla 3, 50134 Florence, Italy; Centro di Salute Globale, Regione Toscana, Via Gaetano Pieraccini 24, 50141 Florence, Italy; Centro per le Malattie Tropicali, Ospedale Classificato Equiparato “Sacro Cuore Don Calabria”, Via Don Sempreboni 5, 37024 Negrar, Verona Italy; Pediatria, Ospedale Classificato Equiparato “Sacro Cuore Don Calabria”, Via Don Sempreboni 5, 37024 Negrar, Verona Italy; ISGlobal, Barcelona Ctr. Int. Health Res. (CRESIB), Hospital Clínic, Universitat de Barcelona, Carrer Rosselló 132, E-08036 Barcelona, Spain; Sezione di Pediatria, Dipartimento di Scienze Della Salute, Università degli Studi di Firenze, SODc Malattie Infettive, Viale Pieraccini 24, 50139 Florence, Italy; Dipartimento Attività Integrate di Pediatria Internistica, Azienda Ospedaliero Univeristaria Meyer, Viale Pieraccini 24, 50139 Florence, Italy; SOD Malattie Infettive e Tropicali, Azienda Ospedaliero-Univeristaria Careggi, Largo Brambilla 3, 50134 Florence, Italy

**Keywords:** Neurocysticercosis, Cysticercosis, Europe, Epilepsy, Italy, Spain

## Abstract

In Europe the management of neurocysticercosis (NCC) is challenging because health care providers are unaware of this condition, thus leading to diagnostic delay and mismanagement. The aim of this study is to retrospectively review the cases of NCC observed in five centers located in Florence, Negrar (Italy) and Barcelona (Spain). A total of 81 subjects with NCC were evaluated in the period 1980–2013. By applying the Del Brutto’s criteria 39 cases (48.1 %) were classified as definitive cases, 31 (38.8 %) as probable cases and 11 (13.6 %) did not satisfy the diagnostic criteria. Continent of origin was known for 80 subjects. Latin America and Asia were the most frequent continents of origin (*n* = 37; 46.3 % and *n* = 22; 27.5 %) followed by Europe (*n* = 14; 17.5 %) and Africa (*n* = 7; 8.8 %). Compared with adults, paediatric patients were more likely to have eosinophilia, to have other parasitic infections, to be asymptomatic, to not be treated with antiepileptic drugs or analgesic and to heal. The study shows that there are some hurdles in the management of NCC in Europe. A not negligible portion of patients diagnosed at reference centers do not fully satisfy Del Brutto’s diagnostic criteria. The higher portion of asymptomatic subjects found among the paediatric group is probably related to an ongoing serological screening among adopted children coming from endemic regions. The value of such a serological screening should be better assessed by a further cost-effective analysis.

**Electronic supplementary material:**

The online version of this article (doi:10.1007/s10072-016-2606-x) contains supplementary material, which is available to authorized users.

## Introduction

Cysticercosis/Taeniasis is listed among the so called Neglected Tropical Diseases (NTD), which are a group of 17 diseases with distinct characteristics that thrive mainly among the poorest populations [1]. Even though one of the classical features of NTD is that they were restricted to tropical and subtropical areas [2], the phenomenon of international travel and migration make possible to observe these diseases in temperate areas such as Europe [3]. Epidemiological data indicate that in Europe we are currently observing the overlapping between autochthonous cases that are disappearing, and imported cases that are rising as a consequence of travels and migrations [4].

The management of cysticercosis, and especially of neurocysticercosis (NCC), the central nervous system localization of the disease, is challenging in contexts where the disease is not frequent and health care providers may be unaware of this condition, thus leading to diagnostic delay and mismanagement [4]. The aim of this study was to review the cases of cysticercosis observed in five Southern European tropical diseases centers and to compare the characteristic of pediatric (<15 years) and adult patients.

## Materials and methods

This is a retrospective observational study on cases of NCC observed in five European centres located in 3 cities. The participating centres are the Centre for Tropical Diseases and the Paediatric department of Sacro Cuore-Don Calabria Hospital, Negrar (Italy), the Infectious and Tropical Diseases Unit, Azienda Ospedaliero-Universitaria Careggi (AOUC), Florence (Italy), the Department for International Adoptees and Immigrant Children, Infectious Diseases Unit, Meyer Pediatric Hospital, Florence (Italy) and the Hospital Clinic, Barcelona (Spain). All the five centres are infectious diseases centres with inpatient and outpatient facilities and services especially dedicated to the study and management of tropical diseases in non endemic context. The two pediatric centres offer a screening service for adopted children for several infectious diseases including a serological screening for cysticercosis as recommended by an Italian protocol [5].

Clinical records of all patients with a diagnosis of cysticercosis managed in the period 1980–2013 in everyone of the participating centres were retrospectively reviewed. The cases were retrieved with the help of clinical and lab logs which are routinely compiled in each centres. The diagnostic criteria proposed by Del Brutto et al. (Table 1, [6]) have been retrospectively applied to each case.

**Table 1:** Diagnostic criteria for neurocysticercosis according to Del Brutto et al. [6]

Absolute	1. Histological demonstration of the parasite from biopsy of a brain or spinal cord lesion 2. Cystic lesions showing the scolex on CT or MRI 3. Direct visualization of subretinal parasites by funduscopic examination
Major	1. Lesions highly suggestive of neurocysticercosis on neuroimaging studies^a^ 2. Positive serum EITB^b^ for the detection of anticysticercal antibodies 3. Resolution of intracranial cystic lesions after therapy with albendazole or praziquantel 4. Spontaneous resolution of small single enhancing lesions^c^
Minor	1. Lesions compatible with neurocysticercosis on neuroimaging studies^d^ 2. Clinical manifestations suggestive of neurocysticercosis^e^ 3. Positive CSF ELISA for detection of anticysticercal antibodies or cysticercal antigens 4. Cysticercosis outside the CNS^f^
Epidemiological	1. Evidence of a household contact with *Taenia solium* infection 2. Individuals coming from or living in an area where cysticercosis is endemic 3. History of frequent travel to disease endemic areas
Degrees of certainty for the diagnosis of neurocysticercosis	
Definitive	1. Presence of one absolute criterion 2. Presence of two major plus one minor and one epidemiologic criterion
Probable	1. Presence of one major plus two minor criteria 2. Presence of one major plus one minor and one epidemiologic criterion 3. Presence of three minor plus one epidemiologic criterion

The presence of two different lesions highly suggestive of neurocysticercosis on neuroimaging studies should be considered as two major diagnostic criteria. However, positive results in two separate types of antibody detection tests should be interpreted only on the basis of the test falling in the highest category of diagnostic criteria

^a^CT or MRI showing cystic lesions without scolex, enhancing lesions, or typical parenchymal brain calcifications

^b^Enzyme-linked immunoelectrotransfer blot assay using purified extracts of *Taenia solium* antigens, as developed by the Centers for Disease Control and Prevention (Atlanta, GA)

^c^Solitary ring-enhancing lesions measuring less than 20 mm in diameter in patients presenting with seizures, a normal neurologic examination, and no evidence of an active systemic disease

^d^CT or MRI showing hydrocephalus or abnormal enhancement of the leptomeninges, and myelograms showing multiple filling defects in the column of contrast medium

^e^Seizures, focal neurologic signs, intracranial hypertension, and dementia

^f^Histologically confirmed subcutaneous or muscular cysticercosis, plain X-ray films showing “cigar-shaped” soft-tissue calcifications, or direct visualization of cysticerci in the anterior chamber of the eye

*ELISA* enzyme-linked immunosorbent assay

Epidemiological and clinical data of each patient were extracted from the clinical records using an anonymized and standardized Epi Info™, version 3.5, 26th January, 2011. Differences in categorical variables between adult and pediatric cases were assessed using the Chi-square test (*p* values <0.05 were considered significant).

## Results

**Fig. 1:**
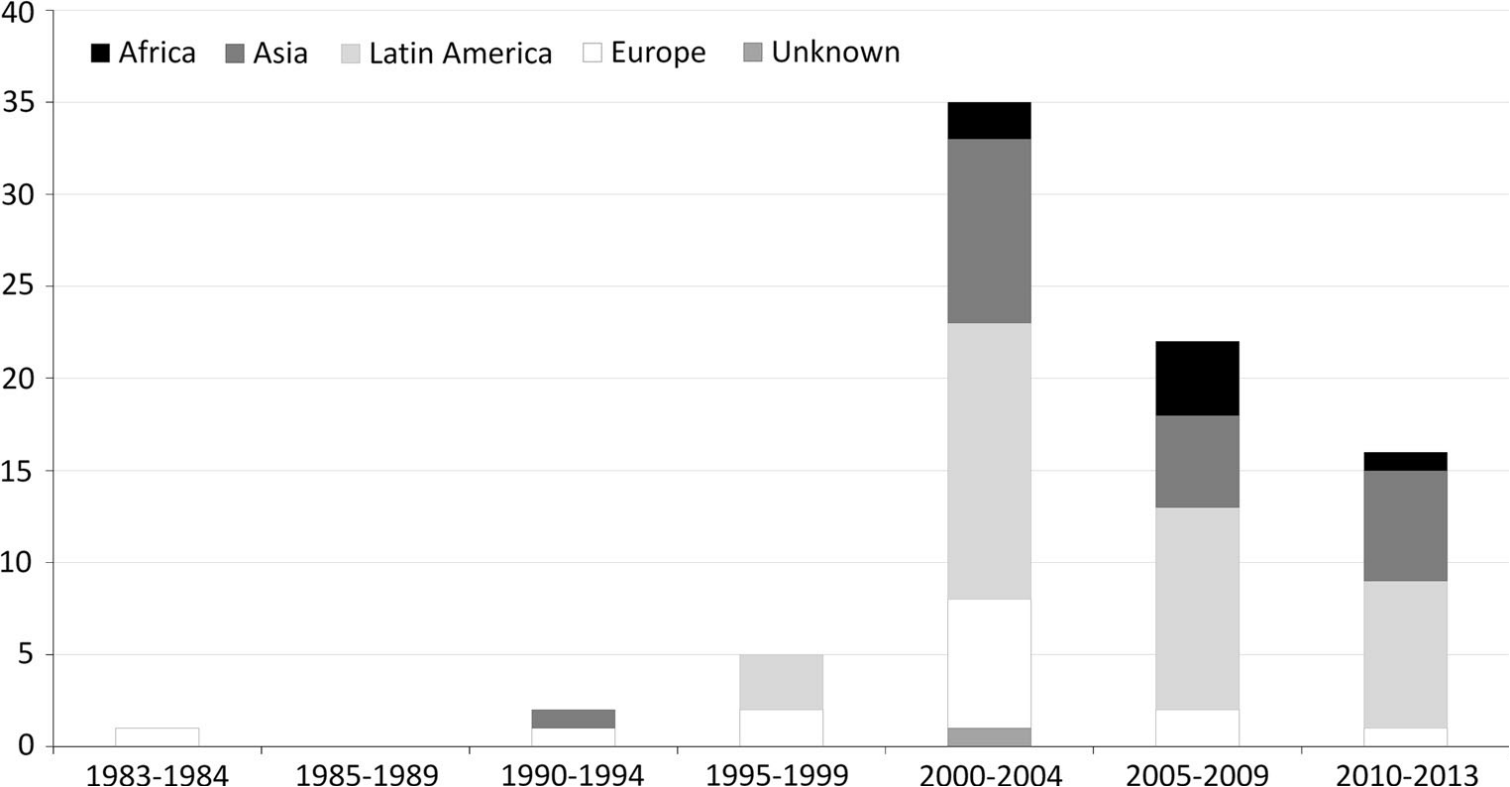
Number of subjects diagnosed with neurocysticercosis by continent of origin and year of diagnosis

Eighty-one subjects with cysticercosis were evaluated at the participating centers in the study period. All patients had central nervous system localization of cysticercosis, while extracerebral lesions were reported only in 1 patient. By applying Del Brutto’s criteria [6] to assess the degree of diagnostic certainty 39 cases (48.1 %) were classified as definitive cases, 31 (38.8 %) as probable cases and 11 (13.6 %) were deemed to be cases of NCC even if the diagnostic criteria were not fully satisfied. All patients underwent at least a brain imaging. In detail 4 (4.9 %) patients underwent CT scan only, 19 (23.4 %) MRI only and 58 (71.6 %) both MRI and CT scan. Table 2 reports the correlation between degree of diagnostic certainty, the number and type of cerebral lesions and the positivity of Enzyme-linked Immunoelectrotransfer Blot (EITB test) on serum. The number of diagnoses by period and continent of origin is reported in Fig. 1. Data on continent and country of origin are reported in Table 3.

**Table 2:** Correlation between the degree of diagnostic certainty according to the Del Brutto’s criteria, number and type of cerebral lesions and the positivity of Enzyme-linked Immunoelectrotransfer Blot on serum in patients with neurocysticercosis

Degree of diagnostic certainty, number and type of cerebral lesions	Rate of positivity of EITB on serum
Single cerebral calcification	3/10 (30 %)
Multiple cerebral calcifications (≥1)	5/13 (38.5 %)
Single non-calcified lesion	5/11 (45.5 %)
Multiple non-calcified lesions (≥1)	12/19 (63.2 %)
Definitive diagnosis	22/31 (70.1 %)
Probable diagnosis	7/26 (26.9 %)
Non-sufficient criteria for diagnosis	5/11 (45.5 %)

**Table 3:** Continent and country of origin of subjects with diagnosis of neurocysticercosis (data available for 80 subjects)

	Number	%
Europe	14	17.5
Italy	7	8.7
Spain	7	8.7
Latin America	37	46.3
Peru	13	19.7
Bolivia	8	12.1
Ecuador	7	10.6
Brazil	3	4.5
Colombia	3	4.5
Dominican Republic	2	2.5
Chile	1	1.5
Asia	22	27.5
India	15	18.7
Cambodia	2	2.5
Vietnam	2	2.5
China	1	1.5
Malaysia	1	1.5
Nepal	1	1.5
Africa	7	8.8
Guinea Bissau	2	2.5
Ghana	1	1.5
Madagascar	1	1.5
Tunisia	1	1.5
Unspecified African countries	2	2.5
Total	80	100

Among the 14 European subjects, 7 patients had a relevant travel history to Latin America (3 cases), Sub-Saharan Africa (2), East European countries (1), or unknown country (1), while 7 cases were considered autochthonous infections acquired in Spain (*n* = 6) or Italy (*n* = 1). Among immigrants and adopted children, the time between the arrival in the host country and the admission for NCC was known in 58 cases. Of those, the majority (*n* = 32; 55.2 %) were admitted for NCC within 1–2 year after arrival, 12 (20.7 %) 3–5 years after arrival, 10 (17.2 %) 6–10 years after arrival and 4 (6.9 %) more than 11 after arrival (maximum delay 18 years).

Considering pediatric patients (*n* = 21), 10 (52.4 %) were males and mean age was 5.8 years (range 1–15 years). Among adult patients evaluated (*n* = 60), 23 (38.3 %) were males and mean age was 37.7 years (range 15–81 years). The mean age of European subjects without history of travel (*n* = 7) was 59.7 years (range 32–81 years). Nine pediatric patients (all asymptomatic and all adopted) were identified on the basis of a serological screening (EITB) and underwent subsequently to a brain MRI study showing lesions compatible with NCC.

Clinical features of patients are reported in Table 4.

**Table 4:** Clinical, serological and radiological features of subjects diagnosed with neurocysticercosis according to the age group (aged <15 years and aged ≥15 years)

	*n* ^a^/*N* ^b^ (%)			
	All (*n* = 81)	Age <15^c^ (*n* = 21)	Age ≥15^c^ (*n* = 60)	*p*
Any symptoms related to cysticercosis	65/75 (86.7)	7/16 (43.7)	55/56 (98.2)	**0.000**
Epilepsy	40/65 (61.5)	6/7 (75)	32/55 (58.2)	0.159
Focal neurological deficit	9/65 (13.8)	0/7 (0)	8/55 (14.5)	0.279
Headache	8/65 (12.3)	0/7 (0)	8/55 (14.5)	0.279
Other neurological symptoms	8/65 (12.3)	1/7 (14.3)	7/55 (12.7)	0.907
Positive EITB on serum	34/68 (50.0)	13/20 (65.0)	21/48 (43.7)	0.110
Positive EIA on serum	2/7 (28.6)	1/3 (33.3)	1/4 (25.0)	0.809
Positive EITB or EIA on serum	36/69 (52.2)	14/20 (70.0)	22/49 (44.9)	0.058
*Taenia solium* taeniasis on parasitological stool examination	1/66 (1.5)	0/20 (0)	1/45 (2.2)	0.501
Positivity for any stool parasite	23^d^/66 (34.8)	11/20 (55)	12/45 (26.7)	**0.027**
Other parasitic infection	19^e^/58 (32.6)	10/19 (52.6)	9/39 (23.1)	**0.024**
Altered “*fundus oculi*” examination	3/34 (88.2)	0/6 (0)	3/27 (11.1)	0.391
Number of CNS lesions detected by brain CT scan and/or MRI				
1	31/69 (44.9)	9/13 (69.2)	21/53 (39.6)	0.054
2–5	20/69 (29)	4/13 (30.8)	14/53 (26.4)	0.752
6–10	10/69 (14.5)	0/13 (0)	10/53 (18.8)	0.089
11–20	5/69 (7.2)	0/13 (0)	5/53 (9.4)	0.249
21–100	3/69 (4.3)	0/13 (0)	3/53 (5.6)	0.379
>100	0/69 (0)	0/13 (0)	0/53 (0)	–
Type of CNS lesions				
Cysts with scolex	4/72 (55.5)	0/15 (0)	4/54 (7.4)	0.295
Calcifications	32/72 (44.4)	9/15 (60)	22/54 (40.7)	0.184
Cysts without scolex	15/72 (20.8)	2/15 (13.3)	13/54 (24.1)	0.372
Enhancing lesions	28/72 (38.9)	6/15 (40)	20/54 (37)	0.824
Hydrocephalus	2/72 (2.8)	0/15 (0)	2/54 (3.7)	0.449
Intraventricular cysts	1/72 (1.4)	0/15 (0)	1/54 (1.8)	0.595
Diagnostic biopsy performed	4/73 (5.5)	0/16 (0)	4/54 (7.4)	0.262
Altered CSF	4^f^/14 (28.6)	0/0 (0)	4/14 (28.6)	–
Altered EEG	13/40 (32.5)	5/7 (71.4)	7/32 (21.9)	**0.010**
Definitive case according to Del Brutto criteria	38/78 (48.7)	8/18 (44.4)	29/57 (50.9)	0.634
Probable case according to Del Brutto criteria	31/78 (39.7)	7/18 (38.9)	22/57 (38.6)	0.982
Del Brutto criteria not satisfied	9/78 (11.1)	3/18 (16.7)	6/57 (10.5)	0.484
Antiparasitic treatment performed^g^	64/77 (83.1)	13/18 (72.2)	50/57 (87.7)	0.117
Albendazole	53/63 (84.1)	13/13 (100)	40/49 (81.6)	0.094
Praziquantel	10/63 (15.9)	0/13 (0)	9/49 (18.4)	0.094
Multiple cycles of antiparasitic treatment	15/64 (23.4)	2/13 (15.4)	13/50 (26.0)	0.423
Treatment with corticosteroids	49/70 (70)	10/18 (55.5)	39/52 (75)	0.120
Treatment with antiepileptic drugs	48/74 (64.9)^h,i^	3/18 (16.7)	43/54 (79.6)	0.000
Treatment with analgesics	11/62 (17.7)	0/18 (0)	11/44 (25)	0.000
Surgical treatment	7/75 (9.3)^l^	0/18	7/56 (12.5)	0.114
Data on clinical follow-up^m^				
Healed	7/33 (21.2)	4/6 (66.7)	3/27 (11.1)	0.013
Improved	15/33 (45.4)	1/6 (16.7)	14/27 (51.9)	0.266
Unchanged	10/33 (30.3)	1/6 (16.7)	9/27 (33.3)	0.754
Worsened	1/33 (3.0)	0/6 (0)	1/27 (3.7)	0.402
Data on radiological follow-up				
Healed	9/55 (16.4)	3/14 (21.4)	6/39 (15.4)	0.605
Improved	25/55 (45.5)	5/14 (35.7)	19/39 (48.7)	0.401
Unchanged	19/55 (34.5)	6/14 (42.9)	12/39 (30.8)	0.412
Worsened	2/55 (3.6)	0/14 (0)	2/39 (5.1)	0.387

Significant *P* values are in bold

^a^Number of subjects with the listed feature

^b^Number of subjects with available information concerning the listed feature

^c^Age was unknown for 3 subjects

^d^15 other protozoa, 3 other helminths, 4 *Hymenolepis nana*, 1 *Taenia solium*

^e^ 5 subjects had 1 parasitic coinfection; 10 subjects had 2 parasitic coinfections; 4 subjects had 3 parasitic coinfections. The diagnosed parasitic coinfections were: 8 strongyloidiasis, 7 schistosomiasis, 6 toxocariasis, 4 ancylostomatidae infestation, 4 trichuriasis, 2 giardiasis, 2 Chagas disease, 1 filariasis due to *Mansonella perstans*, 1 ascariasis, 1 trichinosis, 1 echinococcosis

^f^Increased proteins in 3 cases, increased cells in 1 case

^g^The mean duration of antiparasitic treatments was 23 days (median 28, min 5, max 32)

^h^36 subjects treated with 1 antiepileptic drug, 11 subjects treated with more than 1 antiepileptic drug, 1 subject with no detail on number of antiepileptic drugs

^i^19 subjects treated with phenytoin, 9 subjects treated with valproic acid, 9 subjects treated with carbamazepine, 7 subjects treated with levetiracetam, 6 subjects treated with phenobarbital, 5 subjects treated with oxcarbazepine, 1 subject treated with topiramate, 1 subject treated with lamotrigine, 1 subject treated an unspecified antiepileptic drug

^l^2 cyst excision; 1 ventriculo-peritoneal shunt for hydrocephalus; 1 embolization of left middle cerebral artery aneurysms; 1 hypophysectomy; 1 excision of intraventricular cyst with vetriculostomy; 1 excision of intraventricular cyst with ventriculo-peritoneal shunt

^m^Only symptomatic patients have been included

## Discussion

The study shows several challenges related to the management of NCC in a non endemic area. In this multi-center study, 81 NCC cases were only retrieved in a 33 years period with a peak in the first part of years 2000’, probably due to the massive immigration from Latin American countries observed in that period [7].

The epidemiological characteristics of the patients can help to identify subjects with neurological signs or symptoms that could be affected by NCC. For example, as already reported [4], most NCC cases were imported from Latin America, although cases from every continent have been observed.

This study shows that there are some hurdles in the diagnostic process of NCC. A non negligible portion of patients (13.6 %) with a NCC diagnosis did not fully satisfy Del Brutto’s diagnostic criteria. Moreover, 5.5 % of patients underwent a biopsy-procedure, showing that non invasive tools are not sufficient to achieve the diagnosis of NCC or to rule out a CNS lesion of other origin. In our cases-series, specific serology with EITB method in which many clinicians rely for the diagnosis of NCC is shown to be poorly sensitive with a positivity rate of 70.1 % in cases with “definitive diagnosis”.

In our study pediatric patients were less likely to have symptoms. According to the literature there are several differences among children and adults NCC cases. First of all children are less frequently affected by NCC (Supplementary references a–c) probably due to mechanisms involved in disease acquisition and differences in the reactivity of the immune system against the parasite (Supplementary reference d). Autopsy series from several Mexico City hospitals report a lower prevalence in children if compared with adults (0.5 vs 2 %) [Supplementary references e, f] and asymptomatic NCC detected with MRI is found more often in subjects aged >15 years if compared with those aged <15 years (Supplementary reference b). If compared with children, adult patients with NCC have usually higher incidence of headache (Supplementary reference d), extraparenchimal NCC (Supplementary references d, g), raised intracranial pressure (Supplementary references d, g), altered cerebrospinal fluid findings (Supplementary references d), focal neurodeficits (Supplementary references g–i), depression syndrome (Supplementary references g, j) and seizure recurrence after anticonvulsivants discontinuation (Supplementary references g, k, l). On the other hand children have more frequently seizures (Supplementary reference d), single colloidal parenchymal cysts (Supplementary reference d).

The high portion of asymptomatic cases of NCC in children that we observed is probably related to the ongoing serological screening for cysticercosis among children adopted from endemic regions, which caused a not negligible number of “only” seropositive children to be evaluated for a possible NCC [5].

The screening for cysticercosis in asymptomatic subjects could be theoretically useful in people at risk for NCC in order to early intercept affected subjects and improve their management and clinical outcome. However, there is no clear evidence that treating an asymptomatic subject harboring a live parasite will reduce the probability of neurological symptoms in the future, so the usefulness of a serological screening is questionable.

In conclusion, this study suggest that NCC is a rare condition in Southern Europe, therefore it is not surprising that clinicians may be poorly experienced in the diagnosis and management of it. NCC should be included in the differential diagnosis of patients with neurological signs or symptoms, especially if coming from endemic countries. Available diagnostic tools are not sufficiently accurate in some cases and as a consequence, a definitive diagnosis based on Del Brutto’s criteria cannot always be reached. Moreover, the treatment should be tailored on a case-by-case basis. For this reason, European health care providers might benefit from a transfer of knowledge from colleagues working in endemic areas through the creation of international networks involving expert from endemic areas. The development of a consensus diagnostic procedures and therapeutic decisions would positively impact on the patient care in Europe.

## Electronic supplementary material

Below is the link to the electronic supplementary material.

Supplementary material 1 (DOC 28 kb)

## Abbreviations

Ns: Not significant
EITB: Enzyme-linked immunoelectrotransfer Blot
CNS: Central nervous system
CSF: Cerebrospinal fluid
EEG: Electroencephalogram
EIA: Enzyme immune assay
