# Supplementary material for: Profile of adult and pediatric neurocysticercosis cases observed in five Southern European centers
Source: Neurol Sci. 2016 May 18;37:1349–55. doi: 10.1007/s10072-016-2606-x (PMC4956690; doi:10.1007/s10072-016-2606-x)
Supplement: Supplementary file 1 — Supplementary material 1 (DOC 28 kb) [file 10072_2016_2606_MOESM1_ESM.doc]

Profile of adult and pediatric neurocysticercosis cases observed in five Southern European centers

**Supplementary references**

a. Fleury A, Morales J, Bobes RJ, Dumas M, Yanez O, et al. (2006) An epidemiological study of familial neurocysticercosis in an endemic Mexican community. Trans R Soc Trop Med Hyg 100: 551-558.

b. Prasad KN, Verma A, Srivastava S, Gupta RK, Pandey CM, et al. (2011) An epidemiological study of asymptomatic neurocysticercosis in a pig farming community in northern India. Trans R Soc Trop Med Hyg 105: 531-536.

c. Del Brutto OH (2013) Neurocysticercosis in infants and toddlers: report of seven cases and review of published patients. Pediatr Neurol 48: 432-435.

d. Saenz B, Ruiz-Garcia M, Jimenez E, Hernandez-Aguilar J, Suastegui R, et al. (2006) Neurocysticercosis: clinical, radiologic, and inflammatory differences between children and adults. Pediatr Infect Dis J 25: 801-803.

e. Rabiela M, Lombardo L, Flores F (1972) Cisticercosis cerebral: estudio de 68 casos de autopsia. . Patologıa (Mexico) 27- 40.

f. Sanz CR (1987) Host response in childhood neurocysticercosis. Some pathological aspects. Childs Nerv Syst 3: 206-207.

g. Singhi P, Singhi S (2009) Neurocysticercosis in children. Indian J Pediatr 76: 537-545.

h. Singhi P, Ray M, Singhi S, Khandelwal N (2000) Clinical spectrum of 500 children with neurocysticercosis and response to albendazole therapy. J Child Neurol 15: 207-213.

i. Del Brutto OH, Santibanez R, Noboa CA, Aguirre R, Diaz E, et al. (1992) Epilepsy due to neurocysticercosis: analysis of 203 patients. Neurology 42: 389-392.

j. Forlenza OV, Filho AH, Nobrega JP, dos Ramos Machado L, de Barros NG, et al. (1997) Psychiatric manifestations of neurocysticercosis: a study of 38 patients from a neurology clinic in Brazil. J Neurol Neurosurg Psychiatry 62: 612-616.

k. Ferreira LS, Zanardi VA, Scotoni AE, Li LM, Guerreiro MM (2001) Childhood epilepsy due to neurocysticercosis: a comparative study. Epilepsia 42: 1438-1444.

l. Del Brutto OH (1994) Prognostic factors for seizure recurrence after withdrawal of antiepileptic drugs in patients with neurocysticercosis. Neurology 44: 1706-170
